# Cytokine Release Syndrome Induced by Pembrolizumab for Metastatic Anal Melanoma

**DOI:** 10.1155/crh/5444075

**Published:** 2025-07-01

**Authors:** Erica Martel, Shijia Li, Mayssaa Hoteit, Zubin Bham

**Affiliations:** ^1^Department of Internal Medicine at Bridgeport Hospital, Bridgeport, Connecticut, USA; ^2^Section of Pulmonary, Critical Care at Bridgeport Hospital, Bridgeport, Connecticut, USA

## Abstract

Cytokine release syndrome (CRS) is a rare systemic inflammatory response that can be triggered by certain drugs and infections, commonly diagnosed at a disseminated stage, leading to poor prognosis. This has been well described following chimeric antigen receptor T-cell (CAR-T) therapy but has rarely been reported following antiprogrammed death ligand-1 (PDL-1) therapy. We present the case of an 86-year-old male with metastatic anal melanoma who developed CRS after his 4th cycle of pembrolizumab. His initial presentation was thought to be related to sepsis given his high fevers and hypotension; however, given the lack of improvement despite an extensive workup and broad coverage with antibiotics, CRS was suspected as a potential etiology of his symptoms. Tocilizumab and steroids were successfully used and resulted in the resolution of symptoms without relapse. This case highlights the diagnostic and therapeutic challenges posed by immunotherapy-induced CRS and emphasizes the importance of early recognition to achieve good outcomes.

## 1. Introduction

Mucosal melanoma is usually detected at the advanced stage due to its distinct anatomical location and presents more aggressively with high metastatic potential and dismal prognosis [1, 2]. Due to the rarity of malignant melanoma of the anal canal, there is no standardized best practice established.

Surgery is the cornerstone of the management of nonmetastatic rectal melanoma. In recent years, adjuvant pembrolizumab and nivolumab, a programmed cell death protein 1 (PD-1) inhibitor, have been shown to provide durable antitumor activity in advanced mucosal melanoma by tumor-infiltrating T lymphocytes [3]. While pembrolizumab has revolutionized cancer therapy, it can also cause adverse events. Two rare but serious adverse events triggered by pembrolizumab are cytokine release syndrome (CRS) and hemophagocytic lymphohistiocytosis (HLH) [4]. Although both conditions can present with fever, elevated ferritin, and organ dysfunction, their underlying pathophysiology differs significantly, which has important implications for diagnosis and management.

CRS is typically an adverse effect of immunotherapies which results from an overactivation of T-cells, leading to a cytokine storm, particularly involving cx6, which drives systemic inflammation, multiorgan dysfunction, and potentially shock. The incidence of immune checkpoint inhibitor–induced CRS is estimated at approximately 0.07%. HLH is a hyperinflammatory syndrome characterized by the overactivation of macrophages, T and NK cells, leading to excessive cytokine release, often a genetic or secondary condition with specific diagnostic criteria [5]. Due to the syndromic nature of HLH, the diagnosis can be difficult and is based on the HLH-2004 criteria published by Histiocytic Society and must fulfill five of these eight criteria: fever, splenomegaly, bicytopenia, hypertriglyceridemia and/or hypofibrinogenemia, and hemophagocytosis, low/absent NK-cell-activity, hyperferritinemia, and high soluble interleukin-2-receptor levels.

Given the clinical overlap between these two conditions, it is crucial to distinguish between HLH and CRS for appropriate management. CRS and HLH are both rare hyperinflammatory conditions characterized by excessive cytokine production and are potentially life-threatening. Early recognition and prompt initiation of appropriate treatment are essential to prevent fatal outcomes. While both require rapid intervention, the specific underlying mechanisms and therapeutic approaches differ. Early treatment with intravenous tocilizumab 8 mg/kg often combined with corticosteroids for CRS and HLH is recommended and generally response to treatment is quick. If refractory can consider the addition of etoposide, which is the typical treatment for malignancy-related HLH [6], then for HLH, treatment is adapted to the underlying condition and severity and for refractory cases can consider agents such as anakinra (IL-1 inhibitor), emapalumab (IFN-γ inhibition), and ruxolitinib (JAK inhibitor) [7].

This case highlights the importance of early recognition and prompt treatment of CRS in patients receiving immune checkpoint inhibitors such as pembrolizumab. Although the clinical manifestations of these conditions often overlap, careful evaluation of the underlying pathophysiology and targeted diagnostic workup (e.g., bone marrow biopsy for HLH) can help guide therapy. Corticosteroids, tocilizumab, and supportive care are essential in managing these potentially life-threatening conditions. Clinicians should remain vigilant, particularly in the early stages of pembrolizumab treatment, to identify and treat these serious complications promptly.

## 2. Case Presentation

**Figure 1:**
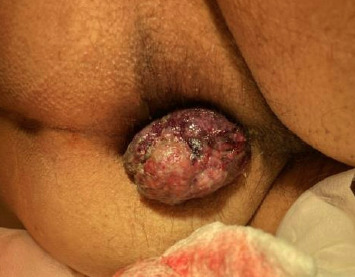
Patient's initial presentation of an anal melanoma lesion.

An 86-year-old male was admitted to a local hospital with fevers, confusion, and weakness for the past two weeks. He had a history of Barrett's esophagus, hypertension, hyperlipidemia, and Type 2 diabetes and was recently diagnosed with anal melanoma (cT2N1M0) after presenting with rectal bleeding and burning discomfort (Figure 1). Anoscopy showed a friable rectal mass, and he underwent complete excision of the mass with negative margins. Pathology revealed a polypoid mass composed of malignant melanoma with ulceration, involving squamocolumnar junctional mucosa positive for SOX10, HMB45, Melan-A, and S100 immunostains and negative for AE1/3 immunostain, supporting the diagnosis. Given that he was deemed to be a poor surgical candidate, oncology recommended palliative pembrolizumab monotherapy, which was administered for 4 cycles over three months. The patient tolerated the initial cycles of therapy well. However, after the second infusion, a possible fever was noted, which resolved following administration of tylenol.

His hospital course was complicated by persistently high-grade non-neutropenic fevers and worsening mental status despite broad-spectrum antibiotics. Extensive sepsis evaluation, including blood cultures, urine cultures, cryptococcal, tick-borne, and CSF studies, was unremarkable. Laboratory tests showed elevated C-reactive protein (CRP) and ferritin levels as shown in Table 1. On Day 4 of admission, he was transferred to the medical intensive care unit (MICU) for hyperthermia, altered mental status, and shock requiring intubation, targeted temperature management, and use of vasopressors with concern for CRS versus HLH versus autoimmune dysregulation in the setting of immunotherapy. He was empirically started on hydrocortisone 100 mg every 8 h and administered tocilizumab (8 mg/kg) empirically for presumed CRS. Ferritin value peaked at 7781 mg/dL making HLH less likely. For HLH, a ferritin level of > 10,000 ng/mL is strongly specific and supports the diagnosis. This threshold is particularly useful in distinguishing HLH from other hyperinflammatory conditions, including CRS. Ferritin levels tend to be much higher in HLH oftentimes > 10,000 ng/mL, which is highly specific for HLH, to help discriminate from CRS [8].

The patient's fever curve and mental status gradually improved over the next 2 days and he was successfully extubated and weaned off vasopressor support. He received an additional dose of tocilizumab and the steroids were gradually tapered with no recurrence of his symptoms.

## 3. Discussion

A diagnosis of CRS is suspected in cases of rapid deterioration following immune checkpoint inhibitors and demonstrates clinical similarities with other hyperinflammatory states such as HLH. Mild cases of CRS are characterized by fever, while severe cases are often characterized by hypotension and multiorgan dysfunction. It can be potentially life-threatening in the setting of bispecific antibody use and T-cell therapies for solid tumors, leukemias, and lymphomas [9]. There are very few reports on immunotherapy-induced CRS with < 0.1% of cases triggered by anti-PD-1 therapy and even rarer, triggered by pembrolizumab. One study showed that ICI-induced CRS was more common in males with advanced cancers [10]. The patient in this case presented with locally advanced anal melanoma who experienced pembrolizumab-induced CRS complicated by multiorgan failure after 4 cycles of immunotherapy. The median time to onset of low and high-grade CRS was 1.3 months (95% CI: 0.23–26.1 months) and 2.5 months (95% CI: 1.35–5.46 months), respectively [11].

ICIs, including anti-CTLA-4 (ipilimumab), anti-PD-1 (pembrolizumab), and anti-PD-L1 antibodies (atezolizumab and durvalumab), function by blocking inhibitory pathways that normally regulate the immune response, thereby boosting T-cell activity against tumors. CRS associated with ICIs arises from excessive immune system activation, leading to the widespread release of inflammatory cytokines such as IL-6, IFN-γ, and TNF-α. This cascade of events results in systemic inflammation, endothelial damage, and potential multiorgan dysfunction. Studies have suggested that individuals with IL-6 gene variants, which lead to overexpression of IL-6 through the trans-signaling pathway, may increase the risk of ICI-induced CRS [12]. Elevated IL-6 levels have been observed in patients experiencing severe CRS, with these heightened levels believed to be linked to T-cell proliferation driven by both therapeutic and inflammatory mechanisms. Studies have shown a direct correlation between cytokine levels and the severity of CRS. Therefore, monitoring cytokine levels in patients can aid in both diagnosing CRS and assessing its severity. The patient in this case showed that four days after receiving tocilizumab, his IL-6 levels increased to 54.9 and 64.4, which is expected to surge briefly after tocilizumab intervention before gradually diminishing.

Treatment of severe CRS, Grade 3 or Grade-4, such as the one that occurred in this patient, can be life-threatening if left untreated and should immediately receive treatment of tocilizumab and high doses of steroids. Usually, significant resolution of CRS-related symptoms is achieved within a few hours after receiving immunosuppressive therapy with steroids and/or tocilizumab, and if no such effect is evident within 8–24 h, a second dose can be given. Other cytokine-targeted therapies have also shown potential in managing the syndrome, including etanercept (TNF-α inhibitor), anakinra (IL-1R antagonist), intravenous immunoglobulin (IVIG), and daclizumab (IL-2R antagonist) [13]. This suggests that clinicians should fully understand the characteristics, clinical manifestations, and treatment principles of CRS and intervene in a timely manner to reduce the adverse risks of medication use. This case also confirms the effectiveness of tocilizumab and high-dose steroids in the treatment of CRS caused by immune checkpoint inhibitors.

## 4. Conclusion

This case highlights an unusual presentation of pembrolizumab-induced CRS in a patient with Stage IV anal melanoma. Early recognition of CRS is important for appropriate treatment and management.

## Figures and Tables

**Table 1:** Essential laboratory data in the hospital course.

Laboratory data	Reference range	On admission (PAT Oh)	ICU admission (PAT 72 h-tocilizumab given)	ICU course (PAT 100 h)
*Blood count*				
WBC 10 ^ 9/L	4–11	10.3	9.3	11.3
Hemoglobin g/dL	13.2–17.1	10.5	8.8	10.4
Band (%)	0.0–1	0.3	0.8	0.8
Seg (%)	39%–72%	78.1	85.3	88
Lymphocyte (%)	17%–50%	12.4	7	7.2
Basophil (%)	0.0–1.4	0.2	0.2	0.1
Eosinophil (%)	0.0–5.0	2.6	4.8	0.3
Platelet (10 ^ 9/L)	150–420	167	137	171

*Coagulation parameters*				
INR	0.9–1.16	1.09	1.14	1.14
D-dimer (mg/L)	< 0.86	—	3.91	—
Fibrinogen (g/L)	187–446	—	680	709

*Biochemistry tests*				
Troponin I (ng/L)	< 10	10	49	—
AST (U/L)	10–35	16	36	32
ALT (U/L)	9–59	14	20	23
Albumin (g/dL)	3.6–5.1	4.8	2.7	2.9
Total bilirubin (mg/dL)	< 1.2	0.3	0.3	0.2
BUN (mg/dL)	8–23	15	18	18
Serum creatinine (mg/dL)	0.4–1.3	0.83	1.01	1.04
Lactate (mmol/L)	0.5–2.2	2.9	2.7	—

*Inflammatory markers*				
CRP (mg/L)	< 1	—	—	> 300
Procalcitonin (μg/L)	< 0.25	—	0.93	—
IL-6 (pg/mL)	< 2	—	54.9	—
Soluble IL-2 receptor (sCD25) pg/mL	175.3–858.2	—	2961	2771.9
Fasting triglycerides (mg/dL)	< 150	—	96	—
Ferritin (ng/dL)	30–400	—	4378	6278

Abbreviations: ALT = alanine serum aminotransferase; AST = aspartate serum aminotransferase; BUN = blood urea nitrogen; CPK = creatine kinase; CRP = C-reactive protein; IL-6 = interleukin-6; INR = international normalized ratio; PAT = postadmission time; WBCs = white blood cells.

## Data Availability

Data sharing is not applicable to this article as no new data were created or analyzed in this study.
